# Neonatal gene therapy effectively prevents disease manifestations in a murine model of Mucopolysaccharidosis type I

**DOI:** 10.1016/j.omtm.2025.101544

**Published:** 2025-07-30

**Authors:** Giada De Ponti, Ludovica Santi, Giorgia Dina, Alice Pievani, Samantha Donsante, Mara Riminucci, Alessandro Corsi, Shaukat Khan, Laura Passerini, Andrea Annoni, Silvia Gregori, Stefania Crippa, Andrea Biondi, Angelo Quattrini, Shunji Tomatsu, Alessandro Aiuti, Maria Ester Bernardo, Marta Serafini

**Affiliations:** 1 San Raffaele Telethon Institute for Gene Therapy - SR-Tiget, IRCCS San Raffaele Scientific Institute, 20132 Milan, Italy; 2 Tettamanti Center, Fondazione IRCCS San Gerardo dei Tintori, 20900 Monza, Italy; 3 Experimental Neuropathology Unit, INSpe, Division of Neuroscience, IRCCS Ospedale San Raffaele, 20132 Milan, Italy; 4 Department of Molecular Medicine, Sapienza University, 00161 Rome, Italy; 5 Department of Biomedical Research, Alfred I. duPont Hospital for Children, Wilmington, DE 19803, USA; 6 Pediatrics, Fondazione IRCCS San Gerardo dei Tintori, 20900 Monza, Italy; 7 School of Medicine and Surgery, University of Milano-Bicocca, 20900 Monza, Italy; 8 Vita-Salute San Raffaele University, 20132 Milan, Italy; 9 Pediatric Immunohematology and Bone Marrow Transplantation Unit, San Raffaele Scientific Institute, 20132 Milan, Italy

**Keywords:** dysostosis multiplex, lentiviral vectors, mucopolysaccharidosis type I, neonatal gene therapy, lysosomal storage disease, Hurler

## Abstract

Mucopolysaccharidosis type I (MPS-I) is a rare pediatric disease caused by mutations in the α-L-iduronidase (*IDUA*) gene encoding for a lysosomal enzyme involved in glycosaminoglycan metabolism. While newborns with the severe Hurler variant are usually asymptomatic at birth, progressive disease manifestations emerge early in life. Since previous studies on lentiviral vector gene therapy (GT) in Hurler patients have demonstrated superior metabolic correction and early beneficial clinical effects, we investigated whether applying this GT approach during the neonatal period could be effective in preventing disease pathology before it becomes irreversible. Thus, newborn MPS-I mice were transplanted with affected bone marrow-derived progenitor cells transduced with an IDUA-encoding lentiviral vector. Treated animals displayed increased IDUA levels, significantly reducing substrate accumulation in analyzed organs, indicating metabolic correction. Skeletal manifestations, typically resistant to conventional therapies, showed improvements at radiographic and histological levels post-treatment. Additionally, a decrease in brain cortex vacuolization and inflammation suggested neurological amelioration. Overall, this study provides a proof of principle demonstrating the effectiveness of neonatal *ex vivo* GT in MPS-I mice and supports its potential for further optimization at the pre-clinical level.

## Graphical abstract

**Figure:**
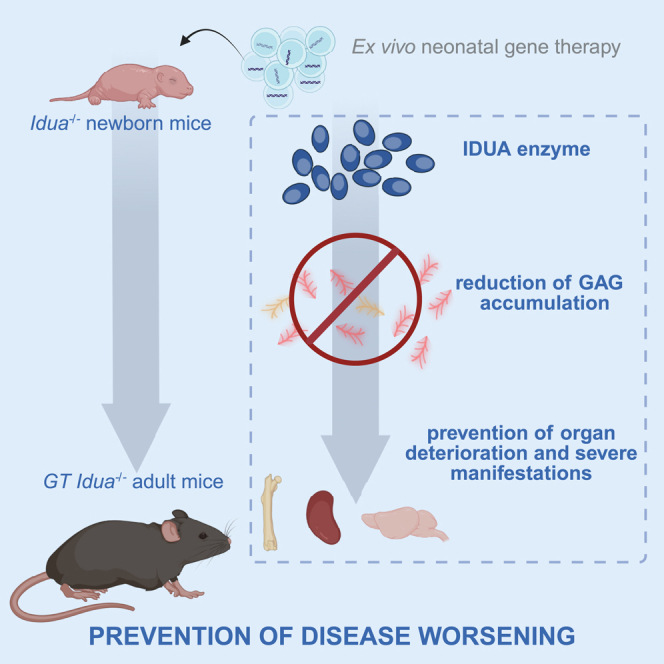

## Teaser

This study provides a proof of principle for neonatal *ex vivo* gene therapy as a potential therapeutic strategy for mucopolysaccharidosis type I. It confirms the effectiveness of this early intervention in preventing disease pathology before it becomes irreversible and poses the base for further refinement and clinical translation.

## Introduction

Mucopolysaccharidosis type I (MPS-I) is an autosomal recessive disease characterized by mutations in the gene encoding for the acid hydrolase α-L-iduronidase (IDUA), which is involved in the lysosomal degradation of dermatan and heparan sulfates (DS and HS). IDUA deficiency is responsible for the intracellular accumulation of glycosaminoglycans (GAGs), causing multisystemic cellular and tissue dysfunction and leading to a broad spectrum of clinical manifestations with a chronic progressive course.^1^ GAGs are sulfated polysaccharide chains that bind core proteins and form proteoglycans, which are key structural components of the extracellular matrix (ECM).^2^ These modulate many cellular processes, including cell differentiation, proliferation, adhesion, migration, and survival, contributing to development and tissue homeostasis. Specifically, DS is found in the corneas, kidneys, epithelium, and both muscular and skeletal tissues, where it contributes to tissue growth. HS is ubiquitously expressed, it plays key roles in the morphogenesis of developing tissues and its accumulation accounts for neurological and skeletal symptoms in MPS-I. The most severe variant of the disorder (Hurler Syndrome, MPS-IH) is characterized by hepatosplenomegaly, respiratory and cardiac anomalies, corneal opacity, reduced auditory acuity, mental retardation, and a spectrum of progressive muscular and skeletal abnormalities (dysostosis multiplex).^2^^,^^3^ Notably, although most newborns with Hurler syndrome show no symptoms at birth, severe manifestations usually emerge early in life.^4^ Timely diagnosis is crucial to preventing irreversible damages, as an early intervention is potentially associated with a better clinical outcome.^5^^,^^6^

Currently, two approved therapies are available to restore functional enzyme activity and treat the main manifestations of MPS-IH: enzyme replacement therapy (ERT), based on weekly intravenous administration, and allogeneic hematopoietic stem cell transplantation (HSCT), which provides a permanent enzyme source through the engraftment of metabolically functional donor cells.^7^ While both treatments effectively reduce GAG storage and improve most visceral manifestations, they exhibit limited efficacy in addressing cardiac valve deformities, skeletal and muscular anomalies, and neurocognitive defects.^2^^,^^8^^,^^9^^,^^10^ These manifestations occur in tissues that are difficult to reach by the enzyme, such as the poorly vascularized/avascular connective tissue or organs isolated from the circulation by specific barriers. Additionally, we have demonstrated that even the combination of these therapies during the neonatal period, although considered the optimal therapeutic window, fails to enhance IDUA activity in murine brains or reduce vacuolated cells and neuroinflammation, with only minimal improvements observed in other difficult-to-treat organs, such as the heart.^11^ Because of the limited efficacy of current therapies, *ex vivo* gene therapy (GT) has emerged as a promising alternative and has been pre-clinically developed and applied to MPS-IH patients, showing superior safety and efficacy compared to HSCT. Studies have shown that gene-corrected hematopoietic stem and progenitor cells (HSPCs) generate circulating and tissue-resident cells capable of releasing supraphysiological levels of the therapeutic enzyme, mediating metabolic cross-correction of non-hematopoietic cells, including those in the brain and skeletal tissues.^12^^,^^13^^,^^14^ Up to now, HSPC-GT has been successfully tested in animal models of lysosomal storage disorders, employing bone marrow (BM)-derived HSPCs that were *ex vivo* cultured, genetically modified, and subsequently transplanted into recipient adult mice to correct difficult-to-treat organs.^12^^,^^15^^,^^16^ In the context of MPS-I, mice transplanted with lentivirus (LV)-transduced affected cells have shown high levels of functional IDUA, leading to better GAG clearance, correction of skeletal abnormalities, and amelioration of neurological symptoms.^13^ Importantly, using autologous cells helps mitigate morbidity and mortality issues associated with allo-immune complications, such as graft failure and graft-versus-host disease, as well as reduces toxicity associated with immunosuppressive treatments.

Solid positive pre-clinical results reported by Visigalli et al. in 2016 demonstrated the absence of significant genotoxicity and tumorigenicity associated with murine HSPCs transduced with LV encoding for *IDUA* (IDUA-LV). These findings paved the way for a phase 1–2 clinical trial for Hurler patients, which is currently ongoing at San Raffaele Hospital (NCT03488394).^17^^,^^18^^,^^19^

To achieve a more effective treatment for MPS-I, it is mandatory not only to enhance enzyme delivery to affected organs, particularly the brain and skeleton, but also to initiate treatment at an early stage of the disease to prevent its onset and progression. Based on this rationale, our study aimed to establish proof of concept for the efficacy of neonatal *ex vivo* GT, by transplanting gene-modified *Idua*^−/−^ BM-derived progenitor cells into newborn *Idua*^−/−^ mice, which exhibit biochemical, metabolic, and morphological defects consistent with the MPS-IH phenotype.^13^^,^^20^ Specifically, we assessed the extent of disease correction compared to untreated MPS-I mice and the normalization compared to wild-type (WT) mice.^13^^,^^20^ We conducted comprehensive analysis, including the measurement of IDUA activity, vector copies/genome (VCN), GAG accumulation, and the presence of antibodies to evaluate the immune response against the IDUA enzyme. Additionally, we evaluated skeletal and neurological correction through radiographs and/or histology, as these organs are difficult to correct using standard methods. Overall, our neonatal GT approach yielded promising results in preventing and correcting the primary disease manifestations of MPS-I, with significant improvements observed at both the skeletal and neurological levels.

## Results

### Early-onset metabolic defects in an MPS-I mouse model

**Figure 1:**
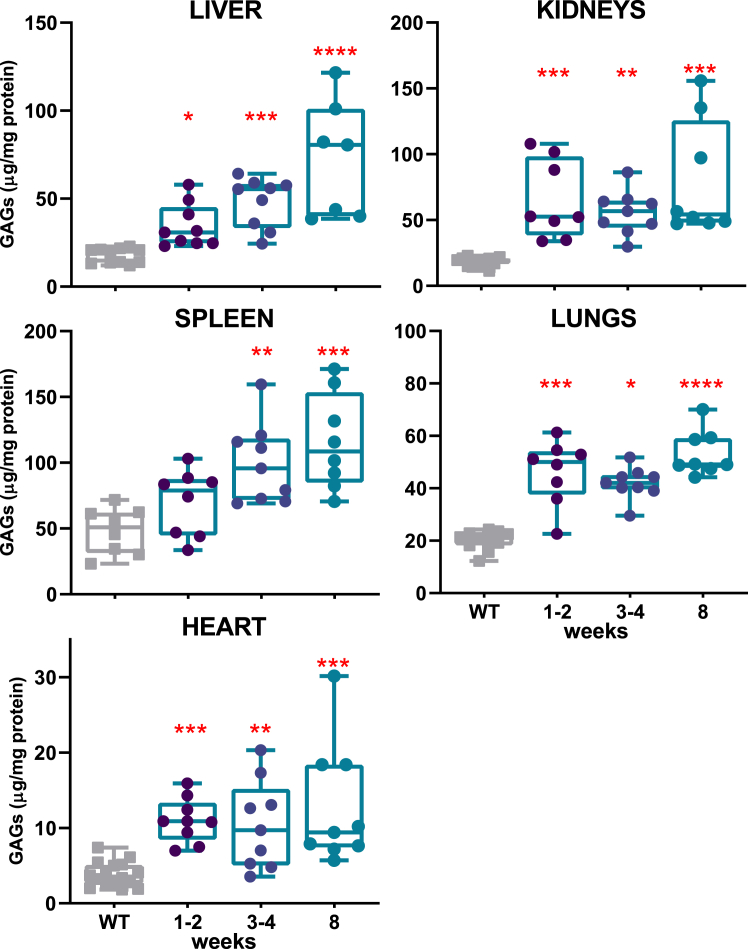
Quantification of GAG accumulation in untreated aging MPS-I mice GAG storage was evaluated in the liver, kidneys, spleen, lungs, and heart of MPS-I mice at 1–2, 3–4, and 8 weeks of age (*n* > 7, for each age). Each data point represents an individual mouse, while bars indicate the median value. WT mice of different ages are represented in gray. ∗*p* ≤ 0.05, ∗∗*p* ≤ 0.01, ∗∗∗*p* ≤ 0.001, and ∗∗∗∗*p* < 0.0001 by non-parametric one-way ANOVA with Kruskal-Wallis test. Red asterisks indicate significance compared to WT mice.

While the MPS-I mouse model has been well characterized in adulthood, little is known about the metabolic consequences of IDUA deficiency during the early postnatal period.^20^ Therefore, we analyzed the GAG accumulation in some affected organs at 1–2 and 3–4 weeks of age, with comparison to adult MPS-I mice (8 weeks old), exhibiting severe symptoms.^21^^,^^22^ As expected, the enzymatic activity was almost null in all the organs, starting from the first weeks of life, and remained nearly undetectable with aging (data not shown). The IDUA deficiency led to a progressive accumulation of GAGs over time, particularly pronounced in the liver and spleen of MPS-I mice compared to WT counterparts (Figure 1). The storage was already detectable from the first weeks of life in all the organs analyzed. In particular, GAG levels were significantly elevated in all visceral organs as early as 1–2 weeks of age compared to WT, except in the spleen, where a significant increase was observed starting from 3 to 4 weeks (*p* = 0.0055). Analysis of GAG accumulation in bone, one of the most challenging organs to treat, revealed a similar pattern of early and progressive accumulation that worsened over time (Figure S1). These findings suggested that the metabolic defect in MPS-I mice emerges early in life, although to a varying extent in different organs, highlighting the potential importance of neonatal treatment to prevent disease progression.

### Optimization of *in vitro* transduction conditions for BM-derived progenitors with IDUA-LV and liquid culture analysis

We used murine progenitor cells isolated from the BM of adult WT mice to determine the optimal transduction condition for IDUA expression that would not alter the cell phenotype and clonogenic potential. *Idua*^−/−^ BM-derived Sca-1 enriched progenitors were transduced with the IDUA-LV vector at several multiplicities of infection (MOIs), ranging from 10 to 80 (Figure S2A). Following transduction, cells were plated for clonogenic analysis and myeloid liquid culture (LC). Cells transduced with MOI values of 10, 20, and 40 showed a similar maintenance of Sca-1 expression, a marker of broader HSPC compartment, over time and no alteration in clonogenic capacity compared to untransduced (UT) cells. A significant reduction in clonogenic capacity was noted at higher MOIs (Figures S2B and S2C). Expanded cells were collected after 14 days of culture and analyzed for IDUA activity and VCN. We found increased IDUA enzymatic activity in transduced cells (range: 21.2- to 50.3-fold) compared to UT healthy cells, with no significant differences among the tested MOIs (*p* > 0.05, Figure S2D), likely due to enzyme saturation. VCN ranged from 0.5 to 6.45 (Figure S2E), but it did not correlate with the levels of enzyme activity achieved. These findings indicate that low doses of the vector are sufficient to induce supraphysiological levels of IDUA without impacting cell phenotype and clonogenic potential. Based on these results, MOI 40 was identified as the optimal infection value for subsequent *in vivo* GT applications.

**Figure 2:**
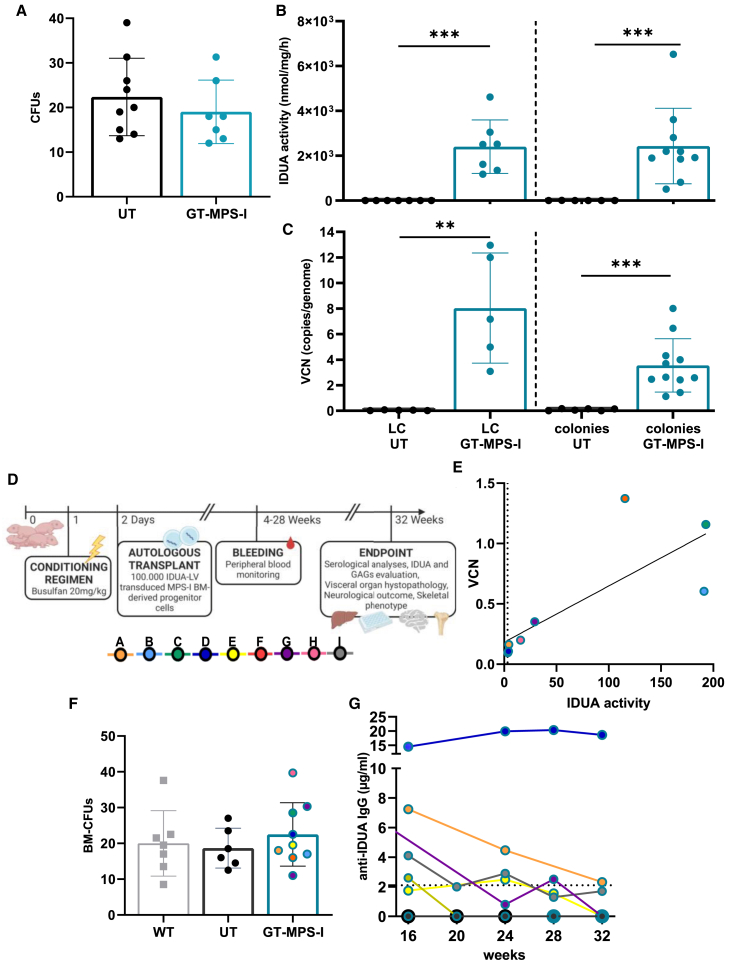
*In vitro* assessment of IDUA-LV transduced BM-derived progenitor cells from MPS-I mice and *in vivo* transplantation (A) Quantification of CFUs generated from BM-derived progenitor cells of MPS-I mice either UT or transduced with the IDUA-LV (GT-MPS-I) at MOI of 40 (*n* ≥ 7). (B and C) IDUA activity and VCN assessed in UT and GT-MPS-I BM cells following LC or CFU-assay (*n* ≥ 5). Each data point represents an individual sample, while the bar indicates the mean value (SD). ∗*p* ≤ 0.01 and ∗∗∗*p* ≤ 0.001 by Mann-Whitney test. (D) Schematic procedure and timeline of the GT approach, with legend of treated mice (A–I). Created in BioRender. Santi, L. (2025) https://BioRender.com/r41t725. (E) Linear correlation between IDUA activity and VCN evaluated over time in treated mice’s PB (*p* ≤ 0.01 by Pearson test). The dashed line represents the median IDUA activity in PB of WT mice (*n* = 8). (F) Number of CFUs formed by BM cells from WT, UT, and GT-treated mice. Each data point represents an individual mouse, while the bar indicates the mean value (SD). *p* > 0.05 by non-parametric one-way ANOVA with Kruskal-Wallis test. (G) Evaluation of antibodies concentration against rhIDUA in GT-treated mice serum monthly until the endpoint (*n* = 9). The dashed line represents the threshold value of positivity.

We then analyzed whether the selected protocol (MOI 40) could be applied to overexpress IDUA in *Idua*^−/−^ BM progenitor cells obtained from adult MPS-I mice, with the aim of reproducing a neonatal *ex vivo* GT approach using genetically modified cells. Also in this setting, both UT and gene-corrected affected cells (GT-MPS-I) retained similar Sca-1 expression (data not shown), as well as proper clonogenic capacity (Figure 2A; *p* > 0.05). Transduced cells exhibited a remarkable increase of IDUA activity compared to UT cells expanded as LC (Figure 2B; range: 1,179.7–4,617.9 nmol/mg/h; *p* = 0.0003). Similarly, colonies, collected as a pool and as a single colony, showed a substantial increase in enzyme activity (range = 511.5–6,520.8 nmol/mg/h; *p* = 0.0002). Additionally, vector copies per genome were assessed, revealing a mean of 8 in LC (range: 3.09–12.9 copies/genome) and a mean of 3.6 in clonogenic progenitors (range: 1.1–8) (Figure 2C; *p* = 0.0079 and *p* = 0.0002, respectively). These results collectively demonstrated the successful transduction of murine *Idua*^−/−^ BM progenitor cells with the IDUA-LV at an MOI of 40, highlighting their potential for transplantation.

### Treatment tolerability over time and elicited immune response

We then refined the protocol for neonatal GT, adapting it from our previous studies (Figure 2D).^5^^,^^6^ Following conditioning with busulfan on day 1 (20 mg/kg; intraperitoneal injection), mice were transplanted on day 2 with 1 × 10^5^ *Idua*^−/−^ BM-derived progenitor cells obtained from MPS-I mice and transduced with IDUA-LV at MOI 40. For our analysis, only GT-treated MPS-I mice with the *Idua*^−/−^ genotype (*n* = 9) were considered, while untreated WT (*n* = 8) and MPS-I (*n* = 6) mice were included as controls.

The GT procedure was well tolerated, with only one intercurrent death recorded in the fourth month post-treatment (Figure S3A). The total body weight of GT-treated animals (median weight 26.2 g [range from 16.9 to 34 g]) did not significantly differ from that of untreated MPS-I mice at 32 weeks (*p* > 0.05). However, a slight body weight decrease of 19.8% was observed, possibly linked to the conditioning regimen-induced toxicity (Figure S3B).^6^ At the time of sacrifice, organ weights were expressed as a percentage of total body weight, revealing a 45.9% decrease in spleen weight (*p* = 0.0294) for GT-treated animals compared to UT MPS-I mice. These results suggest a normalization in the organomegaly associated with the MPS-I mouse model (Figure S3C).

Despite the impossibility of distinguishing engrafted gene-modified cells from recipient cells in our experimental setting, we monitored IDUA activity and VCN parameters over time in the peripheral blood (PB) mononuclear cells of MPS-I mice. Most mice displayed an enzyme activity similar or superior to that measured in WT mice at sacrifice and a linear correlation between VCN and enzyme activity was observed over time (*p* < 0.0215, r = 0.7834; Figure 2E). At sacrifice, we evaluated the proliferative capacity of progenitor cells derived from BM of GT-treated mice using a colony-forming unit (CFU) assay. No significant differences in total colony formation were found between GT-treated and UT MPS-I mice (*p* > 0.05: Figure 2F).

Considering potential specific antibody-mediated effects on treatment efficacy, we assessed the immune response against the IDUA enzyme by detecting antibodies in the serum of treated mice collected from 16 to 32 weeks post-treatment. Notably, most GT-treated mice did not develop supranormal levels of immunoglobulin G (IgG) anti-recombinant human IDUA over time. In contrast, four mice showed a transient antibody response and became tolerant within 8 months after treatment initiation. Only one mouse presented a significantly increased antibody level throughout the study period, with values remaining elevated until the endpoint (Figure 2G). We also assessed the potential immune response to the viral vector by measuring anti-vesicular stomatitis virus glycoprotein (VSVg) IgG levels in the sera of neonatally GT-treated mice, and one-month post-therapy no anti-VSVg IgGs were detected, indicating no immune response to the viral vector (data not shown).

### Metabolic improvement in organs following neonatal GT

**Figure 3:**
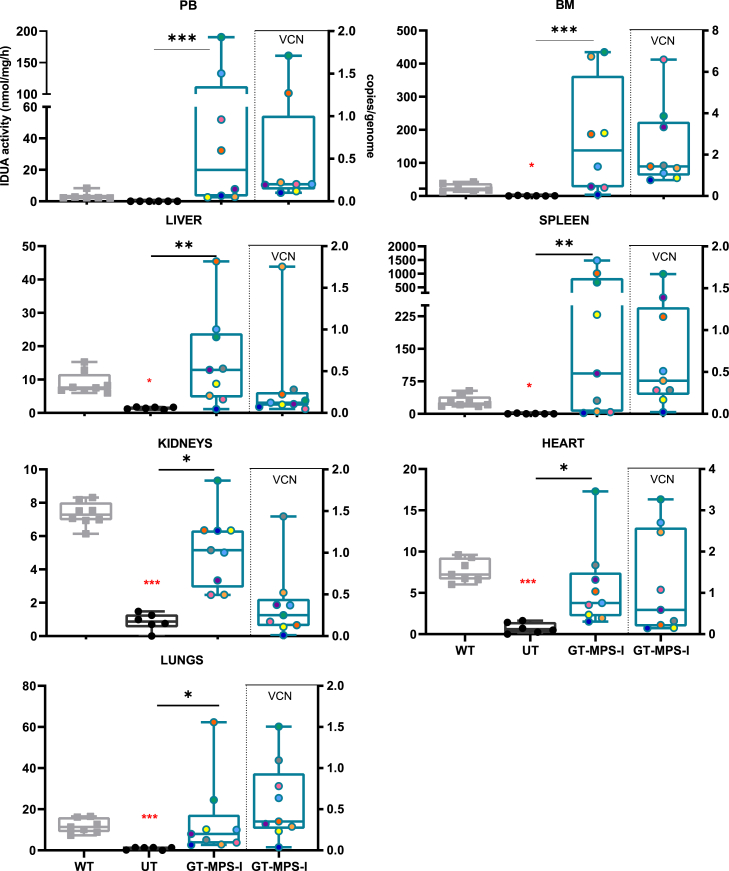
Evaluation of IDUA activity in GT-treated MPS-I mice IDUA activity was measured in the PB, BM, liver, spleen, kidneys, lungs, and heart of wild-type (WT, *n* ≥ 7, represented as gray plots on the graph), MPS-I untreated (UT, *n* = 6, represented as black plots), and GT-treated MPS-I mice (GT-MPS-I, *n* = 8, represented by blue plots) at the study endpoint at 32 weeks of age (left part of the graph). VCN analysis results for the same organs in GT-MPS-I mice are shown on the right side of the graph. Each data point represents an individual mouse, while the bar indicates the median value. ∗*p* ≤ 0.05, ∗∗*p* ≤ 0.01, and ∗∗∗*p* ≤ 0.001 by non-parametric one-way ANOVA with Kruskal-Wallis test. Red asterisks indicate significance compared to WT mice.

We examined the efficacy of the neonatal GT approach in correcting metabolic abnormalities in visceral organs in MPS-I mice. The GT-treated MPS-I group displayed a remarkable increase in IDUA activity compared to untreated affected animals, reaching normal or even supraphysiological levels in most cases (Figure 3, left part of graphs). Specifically, we observed significantly enhanced enzyme activity in PB mononuclear cells (*p* = 0.0003 *vs*. UT MPS-I), BM (*p* = 0.0008), liver (*p* = 0.0063), spleen (*p* = 0.0013), kidneys (*p* = 0.0441), heart (*p* = 0.034), and lungs (*p* = 0.0135). Median IDUA values in all analyzed organs were similar or higher than those in WT mice, with no statistically significant differences between healthy and treated mice.

VCN measurements varied among different GT-treated mice and across organs within the same animal, with an average of 0.5 copies/genome in PB, 2.3 in BM, 0.3 in liver, 0.7 in spleen, 0.4 in kidneys, 1.2 in heart, and 0.6 in lungs (Figure 3, right side of the graph). As expected, VCN in all analyzed tissues of WT animals was null (data not shown).

**Figure 4:**
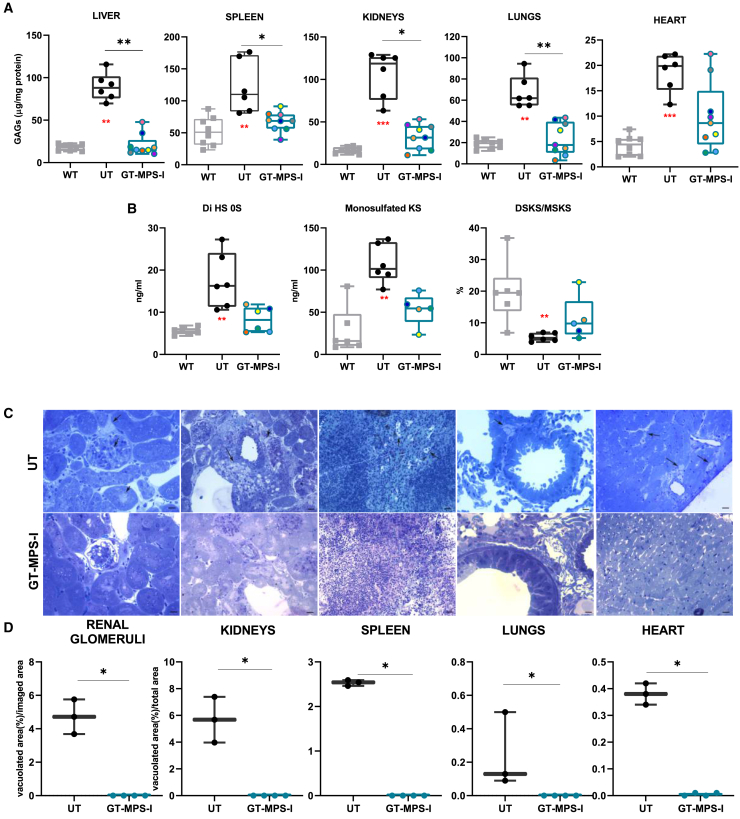
Evaluation of GAG accumulation in GT-treated MPS-I mice (A) Quantification of GAG content in the liver, spleen, kidneys, lungs, and heart of WT mice (*n* = 8, represented as gray plots on the graph), UT MPS-I mice (*n* = 6; represented as black plots), and GT-MPS-I mice (*n* = 9; represented as blue plots) at the study endpoint of 32 weeks of age. (B) Quantification of plasma levels of ΔDiHS-0S, mono-sulfated KS and the ratio of di-sulfated KS over mono-sulfated KS (%DSKS/MSKS) in selected mice at the time of sacrifice (*n* ≥ 5 for each group). ∗*p* ≤ 0.05, ∗∗*p* ≤ 0.01, and ∗∗∗*p* ≤ 0.001 by non-parametric one-way ANOVA with Kruskal-Wallis test. Red asterisks indicate significance compared to WT mice. (C) Representative images of organ sections stained with toluidine blue from UT and GT-treated MPS-I mice at the study endpoint. A 20 μm scale bar was used for kidney, heart, renal glomeruli, and spleen and a 10 μm scale bar for lungs. Black arrows indicate areas of GAG accumulation. (D) Quantification of cytoplasmic vacuolizations in the kidneys (including renal glomeruli), spleen, lungs, and heart of UT (*n* = 3) and GT-treated MPS-I mice (*n* = 4), indicative of distended lysosomes. The percentage of vacuolization was calculated over the total area for organs or over imaged area for renal glomeruli from 10 random fields. Each data point represents an individual mouse, while bars indicate the median value. ∗*p* ≤ 0.01 by Mann-Whitney test.

To evaluate the extent of metabolic correction upon neonatal GT, we measured GAG concentration and cytoplasmic vacuolization in the same visceral organs (Figure 4). Following treatment, GAG quantity was significantly reduced by 77.0% in the liver, 44.6% in the spleen, 69.2% in kidneys, and 65.2% in lungs, compared to UT MPS-I mice, reaching levels similar to those observed in healthy controls. In the heart, treated mice exhibited a 47.2% reduction in GAGs, even though this change was not statistically significant (Figure 4A). Additionally, we perform GAG quantification in plasma, using liquid chromatography triple quadrupole mass spectrometry. Following neonatal GT treatment, mice showed trends toward reduced plasmatic levels of monosulfated keratan sulfate (KS) and ΔDiHS-0S (2-acetamido-2-deoxy-4-O-(4-deoxy-a-L-threo-hex-4-enopyranosyluronic acid)-D-glucose), with a decrease of 37.4% and 52.6%, respectively. Regarding the di- and mono-sulfated KS ratio (DSKS/MSKS), treated mice displayed plasma levels similar to those of WT mice (Figure 4B).

Moreover, we quantified cytoplasmic vacuoles in tissue sections, since these are caused by distended lysosomes and are an indirect marker of GAG accumulation (Figures 4C and 4D). Untreated MPS-I mice exhibited abundant vacuolization in renal glomeruli, kidneys, spleen, lungs, and heart. After GT treatment, vacuoles almost disappeared in all analyzed organs, including the heart (*p* = 0.0286 vs. UT MPS-I mice in all organs), consistent with the reported biochemical improvements (Figure 4D). In summary, the neonatal GT approach demonstrated extensive metabolic correction in the MPS-I mouse model.

### Neonatal GT effects on the brain

**Figure 5:**
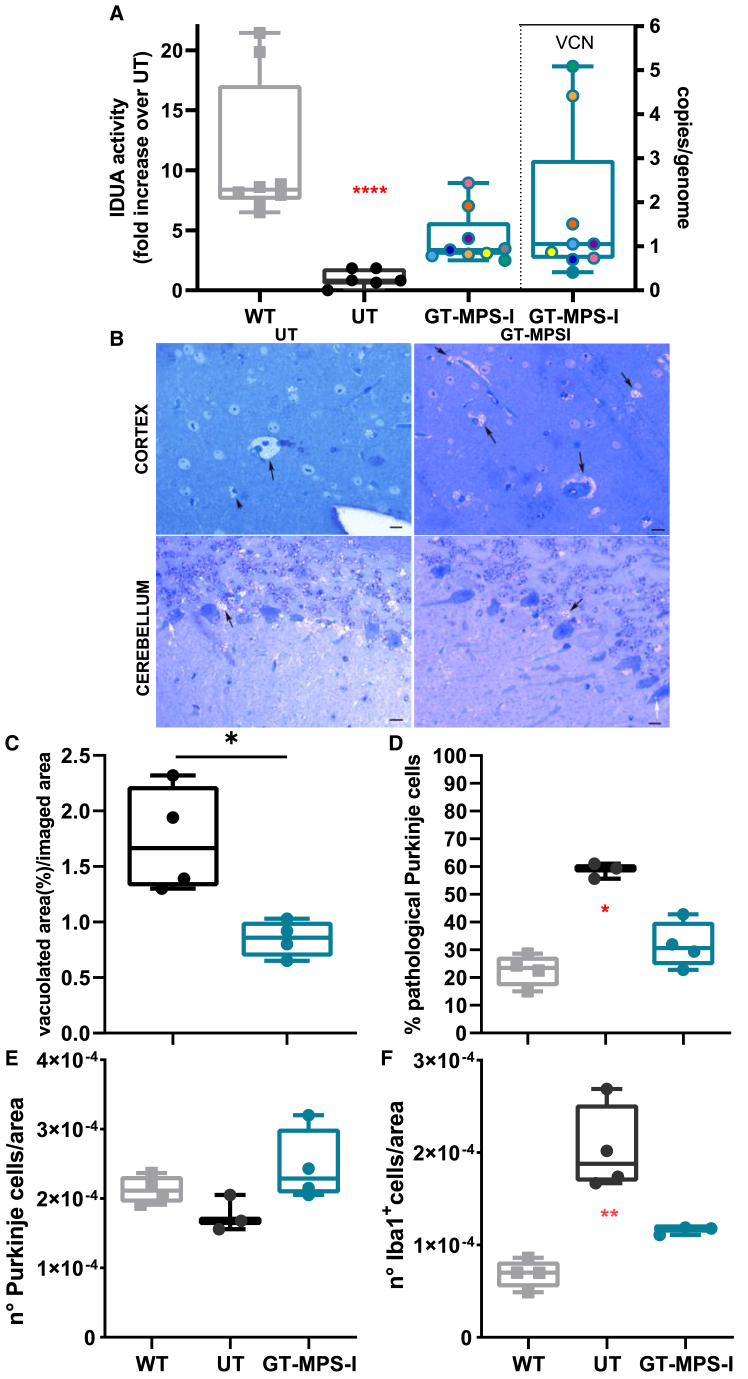
Evaluation of brain disease after neonatal GT (A) Assessment of IDUA activity in the brains of WT (*n* = 8, represented as gray plots on the graph), untreated MPS-I (UT, *n* = 6, represented as black plots), and GT-treated MPS-I mice (*n* = 9, represented as blue plots) at the study endpoint of 32 weeks of age. The IDUA activity was expressed as a fold increase over UT. VCN analysis results are indicated on the right side of the graph. (B) Representative images of toluidine blue-stained cerebral sections from the cortex and cerebellum (Purkinje cell layer) of untreated and GT-treated MPS-I mice at the study endpoint. A 10-μm scale bar was used as a reference for the images. White arrows indicate storage inside the Purkinje cells, while black arrows highlight GAG deposition outside the cells. Ten images from 3 to 4 different mice per group were quantified. (C) Quantitative analysis of vacuolization in the cortex of untreated and GT-treated MPS-I mice, expressed as the percentage of vacuolation relative to the imaged area (*n* = 4) (D and E) Quantification of total Purkinje cells (*n*° cells/area) or pathological Purkinje cells (% of pathological over total Purkinje cells) in WT, untreated and treated MPS-I mice (*n* ≥ 3). (F) Evaluation of neuroinflammation using immunofluorescence, expressed as the number of Iba1^+^ cells per area (*n* ≥ 3). Each data point represents an individual mouse, while bars indicate the median value. ∗*p* ≤ 0.05, ∗∗*p* ≤ 0.01, and ∗∗∗∗*p* < 0.0001 by non-parametric one-way ANOVA with Kruskal-Wallis test. Red asterisks indicate significance compared to WT mice.

Given the limited efficacy of standard-of-care therapies on the central nervous system of MPS-IH patients, we focused our investigation on assessing the impact of neonatal GT on brain function. Following neonatal GT, treated mice showed a 3.9-fold increment of IDUA activity in brain homogenates (*p* = 0.0617 *vs*. UT MPS-I; Figure 5A). Notably, the VCN measured in the brains of treated mice (0.4–5.1 copies/genome) suggested the presence of corrected cells in this organ.

Untreated MPS-I mice exhibited increased vacuolization in the brain cortex compared to healthy mice (1.74% of vacuolization over imaged area; Figure 5B, top). GT treatment ameliorated the disease phenotype, reducing vacuolization by 51.1% (*p* = 0.0286 *vs*. UT MPS-I), approaching WT levels (Figure 5C).

To further elucidate the impact of neonatal GT on brain manifestations, we analyzed the Purkinje cell layer of the cerebellum in treated and control animals (Figure 5B, bottom). Untreated MPS-I mice presented vacuolization due to GAG accumulation both inside (white arrow) and outside (black arrow) Purkinje cells. The percentage of pathological Purkinje cells, identified based on their altered morphology, including changes in shape and content, decreased by 45.9% after neonatal GT (Figure 5D), with no significant changes in the total number of Purkinje cells per area (Figure 5E). As neuroinflammation is implicated in MPS-I brain pathology, we evaluated the density of ionized calcium-binding adaptor molecule 1 (Iba1)-positive cells within the microglia in the brain, indicating microglial activation. A 2.9-fold increase in Iba1^+^ cells was observed in untreated MPS-I, while GT-treated MPS-I mice displayed a 42.9% reduction in microglia activation, approaching WT values (Figure 5F).

### Skeletal phenotype following neonatal GT

**Figure 6:**
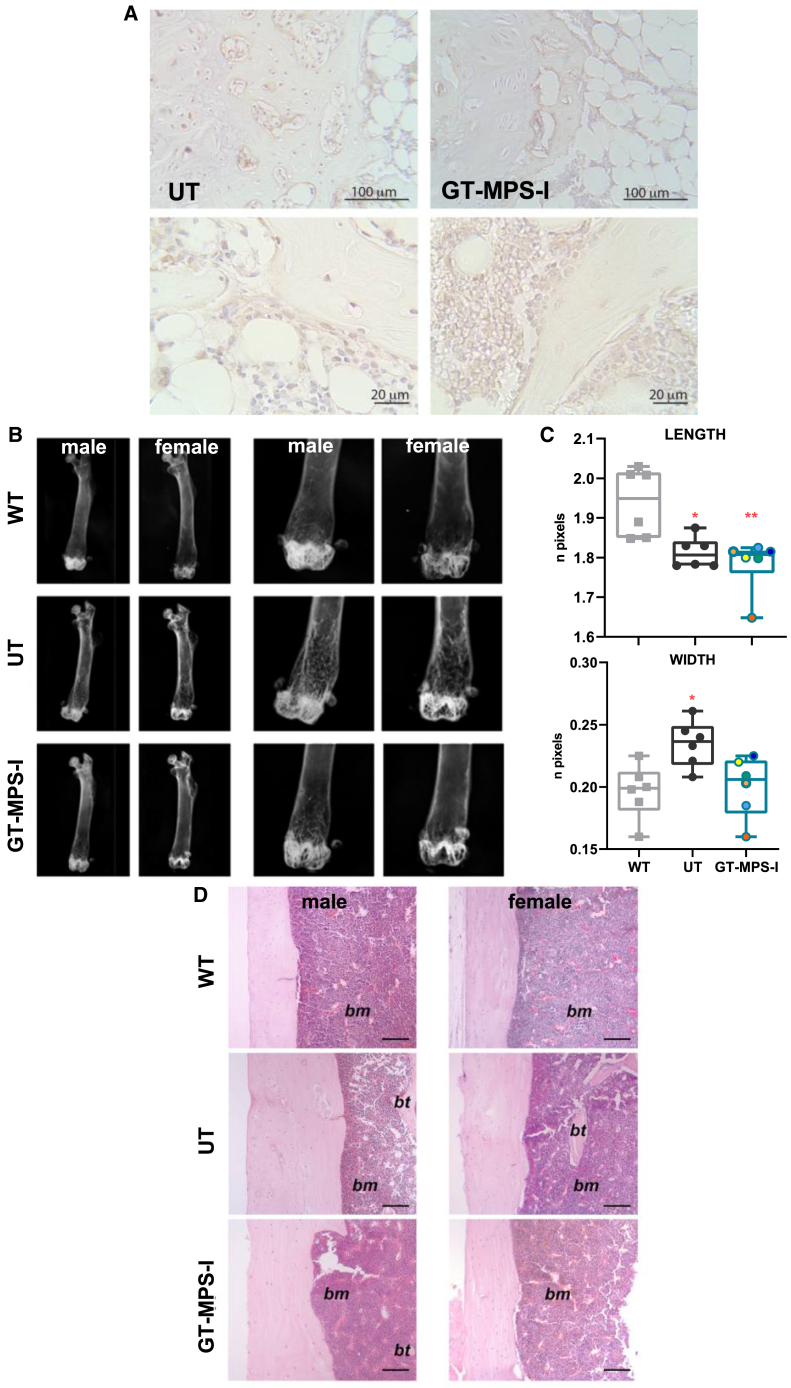
Bone alterations amelioration in neonatal GT-treated mice (A) Representative images of immunostaining on femurs to detect HS accumulation in untreated MPS-I (UT) and GT-treated MPS-I (GT-MPS-I) mice at study endpoint (scale bars, 100 μm and 20 μm). (B) Representative radiographic images of femurs from WT, UT, and GT-MPS-I mice at the study endpoints of 32 weeks. (C) Analysis of femur length and mid-length width in both male and female mice among different groups (*n* = 6 for each group). Each data point represents an individual mouse, while bars indicate the median value. (D) Representative histological images of femur bone cortex (magnification 10×, hematoxylin and eosin stain) from male and female WT, UT, and GT-MPS-I mice at the study endpoint. A 100 μm scale bar was used. bt, bone trabeculae; bm, bone marrow. ∗*p* ≤ 0.05 and ∗∗*p* ≤ 0.01 by non-parametric one-way ANOVA with Kruskal-Wallis test. Red asterisks indicate significance compared to WT mice.

We thoroughly assessed the skeletal outcomes of neonatal GT-treated MPS-I mice, as the bone represents another hard-to-treat tissue for current therapies. Given that HS is one of the GAGs specifically accumulated in MPS-I, we performed an immunostaining on bone samples to confirm reduced HS accumulation after neonatal GT in this compartment (Figure 6A). Neonatal GT-treated mice displayed a marked reduction in HS signal in both the bone and the growth plate.

Radiographic measurements of mouse femurs revealed a 6.5% reduction in length and a 19.4% increase in mid-length width in untreated MPS-I compared to WT mice (*p* = 0.0218 and *p* = 0.0187, respectively). Neonatal GT-treatment resulted in a 14.6% reduction in femur width, with no significant changes observed in its length (Figures 6B and 6C).

**Figure 7:**
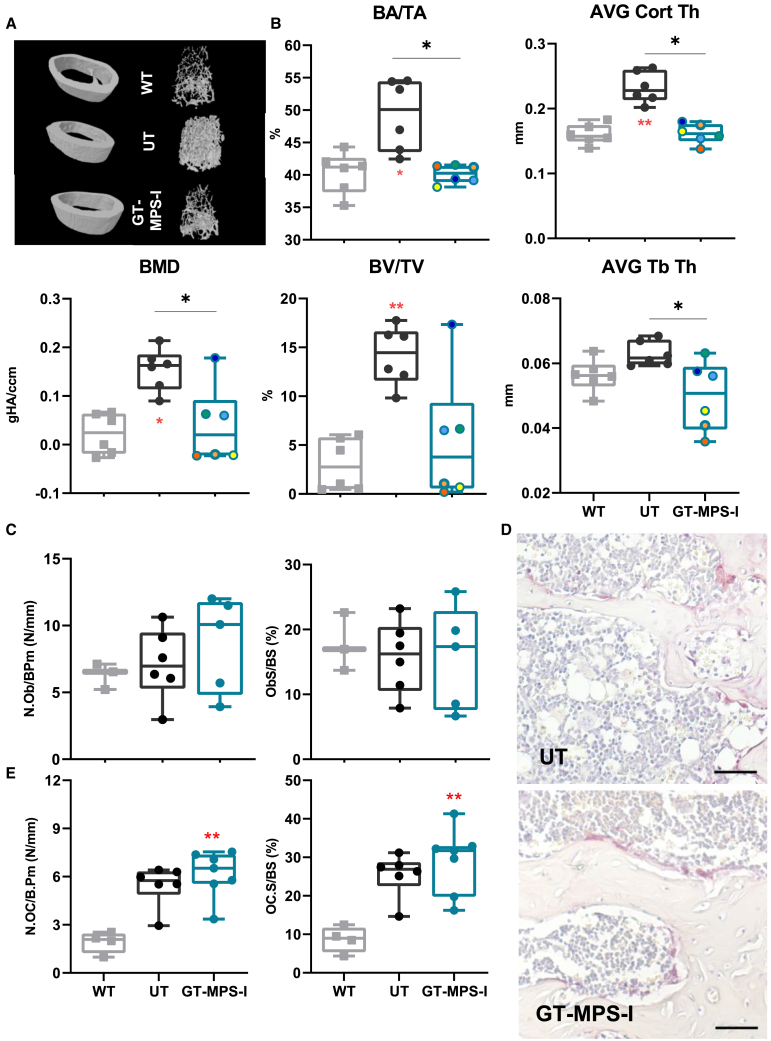
Micro-CT evaluation of the femur architecture after neonatal GT (A) Representative images of cortical (left) and trabecular (right) volumetric models reconstructed from micro-CT scans from WT, untreated MPS-I (UT), and GT-treated MPS-I (GT-MPS-I) mice at the study endpoint of 32 weeks. (B) Evaluation of trabecular and cortical bone morphometrics in femurs from different groups. Bone area over total area (BA/TA) and average cortical thickness (AVG Cort Thick) were measured in the cortical portion, while bone mineral density (BMD), bone volume over total volume (BV/TV), and average trabecular thickness (AVG Tb Th) were analyzed at the trabecular level (*n* = 6 for both groups). (C) Graph of osteoblast number per bone perimeter (N.Ob/B.Pm, N/mm) and osteoblast surface per bone surface (Ob.S/BS, %) (*n* ≥ 3). (D) Representative histological images of TRAP positive osteoclasts (red stain; scale bars, 50 μm) performed on femurs from UT and GT-MP-SI mice at the study endpoint. (E) Graph of osteoclast number per bone perimeter (N.Oc/B.Pm, N/mm) and osteoclast surface per bone surface (OC.S/BS, %) (*n* ≥ 4). Each data point represents an individual mouse, while bars indicate the median value. ∗*p* ≤ 0.05 and ∗∗*p* ≤ 0.01 by non-parametric one-way ANOVA with Kruskal-Wallis test. Red asterisks indicate significance compared to WT mice.

Histopathological analysis showed cortical bone thickening in the femurs of untreated MPS-I mice compared to WT controls, which was clearly reduced in GT-treated MPS-I mice (Figure 6D). This finding was quantitatively proven by micro-computed tomography (micro-CT, Figure 7A) imaging, which revealed significant alterations in several trabecular and cortical bone parameters in untreated MPS-I compared to WT mice, with most of these changes almost completely normalized in neonatal GT-treated mice (Figures 7A and 7B). Indeed, the bone phenotype of MPS-I mice was characterized by an overall increase in bone area fraction (BA/TA; *p* = 0.0174), bone volume fraction (BV/TV; *p* = 0.0083), bone mineral density (BMD; *p* = 0.0174), and cortical thickness (*p* = 0.0059) in comparison with WT animals (Figure 7B). Neonatal GT-treated mice displayed a marked reduction in all these parameters, with no significant differences compared to WT values (Figure 7B). Interestingly, the high bone mass phenotype is probably not imputable to increased osteoblast (Ob)-mediated bone formation, as the osteoblast number per bone perimeter (N.Ob/B.Pm) and the osteoblast bone surface (ObS/BS) were similar between untreated MPS-I and WT mice, and remained unaltered after neonatal GT (Figure 7C). Conversely, the osteoclast number (N.Oc/B.Pm) and osteoclast surface (Oc.S/BS) were increased by 2.8- and 2.9-fold, respectively, in untreated MPS-I compared to WT mice, and neonatal GT-treatment did not result in statistically significant decreased osteoclastogenesis (Figures 7D and 7E). Collectively, these data suggest that the neonatal GT appears effective to maintain both the bone structure and remodeling activity, thus preventing the development and progression of the skeletal abnormalities observed in MPS-IH patients.

## Discussion

MPS-IH is a severe infantile disease that highly impairs the young patients’ quality of life and lifespan. Without treatment, survival is less than 10 years on average and the diagnosis is often delayed due to vagueness of symptoms.^23^ The current standard of care for this disease, represented by HSCT, does not completely address manifestations at the level of the central nervous system (CNS), skeleton, heart, and eye, leaving patients with a significant residual disease burden. In addition, supportive interventions and ERT are often needed to compensate for the incomplete correction of these difficult-to-treat disease manifestations.^24^^,^^25^

*Ex vivo* GT has recently emerged as a promising alternative treatment option for MPS-IH patients, and a phase 1–2 clinical trial, based on LV-GT, is currently ongoing at San Raffaele Hospital (NCT03488394),^17^^,^^18^^,^^19^ showing extensive metabolic correction and initial clinical responses at systemic level, including the CNS and skeletal system.

As previously demonstrated by our group and others, it is evident that initiating intervention before irreversible disease damages occur is pivotal for achieving optimal outcomes, especially in terms of skeletal recovery.^5^^,^^6^^,^^10^ Indeed, a critical parameter to achieve successful cell and GT in MPS-I is the ability to intervene early enough, so that the disease severity has not irreversibly compromised the patient. In line with this, both direct *in vivo* administration of viral vectors or gene editing strategies have been previously tested in MPS-I mice, highlighting the advantages of a precocious intervention.^26^^,^^27^^,^^28^^,^^29^ Early direct administration of viral vectors in MPS-I neonate mice has been explored using both LVs, adeno-associated viruses (AAVs) and gammaretroviral vectors reporting promising results.^26^^,^^27^^,^^30^ Considering the paramount importance of early treatment, the primary purpose of this project was to establish a proof of concept for the efficacy of neonatal *ex vivo* GT approach, specifically in preventing symptoms that are typically refractory to correction, i.e., dysostosis multiplex and CNS manifestations. Using a well-characterized *Idua*^−/−^ mouse model,^13^^,^^20^ which exhibits biochemical, metabolic, and morphological defects in adulthood consistent with the disease phenotype observed in MPS-IH patients, we found that the progression of the disease manifests early in life. Metabolic analyses of visceral organs at various time points suggested that the deficiency in IDUA enzyme led to the accumulation of undegraded substrates as early as the first weeks of life, although to a varying extent in different organs, supporting the importance of an early treatment approach.

First of all, we optimized the conditions for the LV infection of murine *Idua*^−/−^ BM-derived Sca-1 enriched progenitors, allowing for efficient transduction and a significant increase in IDUA activity without compromising the proliferating capability of primitive cells, needed for successful engraftment in newborn recipients. Although Sca-1 is widely used as a marker to identify stem and progenitor cells, it is not completely specific. For this reason, the Sca-1 enriched cells used in our setting may contain a mixture of stem cells and more differentiated cells.

The use of cells obtained from CD45.2 affected mice, without the insertion of a cell tracking protein, transplanted in CD45.2 matched recipients limited our ability to discriminate between effective engraftment of donor cells and recipient ones. Nonetheless, the presence of the enzyme monitored over time in the PB, together with its linear correlation with vector integration, provided evidence of IDUA release from gene-modified engrafted cells, starting as early as 4 weeks after transplantation. The evaluation of donor hematopoietic cell chimerism was not included in this study and this could represent a weakness, as the experimental design prioritized the assessment of therapeutic benefit through clinical and biochemical endpoints. The correlation between the engraftment level and these parameters could ameliorate the interpretation of the analyses, since in our settings we can only speculate about the transplanted cell engraftment through the evaluation of disease parameters. Concerning the *ex vivo* characterization of the engrafted hematopoiesis, we were able to ascertain that the number of CFUs obtained from the BM of GT-treated mice was comparable to that of control animals, suggesting that our approach did not impair progenitor cell function. In line with this, previous clinical trials using LV-transduced HSPCs have demonstrated successful reconstitution of the hematopoietic compartment, with long-term survival and maintenance of polyclonal, gene-corrected progeny.^18^^,^^31^^,^^32^

Overall, the treatment was well tolerated, with almost all MPS-I mice that received the GT surviving from weaning to the endpoint. We monitored the formation of anti-IDUA antibodies, as the immune response against IDUA, especially during ERT, may potentially affect therapeutic efficacy.^33^^,^^34^ Among the GT-treated mice, only one developed an immune response, which, in line with previous findings of Dickson et al., partially reduced therapeutic efficacy.^35^ Differently, the few mice that exhibited transient low immunization displayed improvements in disease manifestations, with variable results depending on the organ considered. Although early administration of GT may promote immune tolerance to the exogenous enzyme, the myeloablative conditioning with busulfan can still trigger antigen-specific immune responses, a process further favored by the inflammatory environment induced. Considering the biochemical parameters, in our previous works, we observed only a partial increase in IDUA activity in visceral organs after neonatal administration of different treatments, even in combination, which was nonetheless sufficient for some amelioration of disease manifestations.^5^^,^^6^^,^^11^ Notably, a neonatal approach consisting of transplantation of donor’s cells and life-long injections of ERT, despite the metabolic improvement, would represent a significant burden on patients’ quality of life. Instead, in this case, after one single procedure of GT, mice achieved normalization of IDUA activity in all visceral organs and increased levels in many compartments. The IDUA recovery led to almost complete GAG clearance in visceral organs of GT-treated mice. Spleen, lungs, liver, and kidneys of the treated group achieved full metabolic correction, with no differences in GAG quantity and cytoplasmic vacuolization compared to healthy mice. Even the structure of renal glomeruli, impaired by GAG storage in untreated MPS-I mice, was rescued. Despite a significant decrease, the heart was the only organ in which the GAG levels remained slightly higher than those of WT mice. However, histopathological analyses suggested that myocardial fibers were completely free of evident vacuolization in treated mice. Vacuolization, resulting from lysosomal accumulation and distension, is considered a marker of disease progression and its reduction following treatment indirectly indicates an improvement in metabolic function. Of note, all neonatal treatments previously tested by our group in the MPS-I mouse model, including the combination of HSCT and ERT, were not able to induce full histological correction of the heart.^11^ Differently from Pompe disease, which responds well to treatment, the specific pathological changes in the heart of MPS-I, including the dense and fibrotic ECM and the multifocal cardiac impairment, pose a significant barrier to achieving complete correction.^36^^,^^37^

While we observed a linear correlation between IDUA activity and VCN in PB of treated mice over time, overall, the vector copies analyzed in the different organs were not similarly correlated with enzymatic activity and we did not identify a VCN value associated with the metabolic correction. In terms of GAG storage and VCN, we detected a trend of negative correlation in the heart and spleen. Surprisingly, the VCN in the heart was higher than the average spleen VCN. Despite no explanation being directly linked to this increase compared to this hematopoietic organ, this cardiac value was lower than the VCN in the BM, suggesting no defined effect on the population dimension in the organ to the final achieved VCN. Overall, the VCN values achieved in our study did not exceed one target gene copy, ideally minimizing the already low risk of insertional mutagenesis associated with the use of LVs.^38^^,^^39^^,^^40^ Moreover, in this work, we used the same LV backbone employed in the metachromatic leukodystrophy (MLD) and MPS-IH clinical trials. In these studies, a stable and highly polyclonal repertoire was found in PB and BM samples obtained from patients treated with HSPC-GT, without dominant clones or notable enrichment for oncogenes or tumor-suppressor genes.^18^^,^^32^ Similar integration profiling data have been reported in other studies of LV-based GT.^41^^,^^42^^,^^43^ Given that the integration site is primarily determined by the LV backbone, independent of the specific transgene incorporated, we believe the risk of genotoxicity associated with the proposed GT approach is minimal.

The GT approach successfully normalized plasmatic levels of ΔDiHS-0S and monosulfated KS in mice, which are known indicators of clinical and skeletal severity, respectively.^44^^,^^45^ These findings were consistent with the skeletal rescue observed in GT-treated mice. Importantly, it has been proposed that increased KS levels in blood observed in MPS patients, together with their decrease in the brain, could be associated with CNS damages and subsequent KS release, potentially making KS a biomarker for CNS manifestations.^46^ Nonetheless, further investigations are required to validate this and identify other reliable biomarkers for evaluating therapeutic efficacy in MPSs.^47^

This neonatal GT led to a significant increase in IDUA activity in the brain of affected mice, overcoming the limited effect previously observed for the neonatal HSCT+ERT combination therapy.^11^ The IDUA levels attained in the brain may be adequate to obtain an improvement, as a level of enzyme activity at 15%–20% of normal values may be sufficient to correct CNS manifestations in patients affected with lysosomal storage disorders.^48^ However, it is crucial to consider not only the secretion of the functional enzyme in the brain by GT-corrected progeny of transplanted cells, but also the capacity of other brain-resident affected cells to reuptake it, which could potentially impact therapy efficacy. Alongside, VCN values measured in the brain of neonatally treated mice were comparable to those observed in other GT approaches performed in adult mice, even if contribution from the circulation to the brain VCN could not be excluded, due to lack of saline perfusion before tissue harvesting.^19^ Notably, a reduction in cytoplasmic vacuolization was achieved after neonatal GT in the brain cortex and in the Purkinje cell layer of the cerebellum, which is altered in MPS-I patients and mice.^11^^,^^49^ A similar improvement was not achieved by the neonatal combination therapy. Finally, investigating the impact of treatment on neuroinflammation, which has been implicated in MPS-I pathology, revealed normalization of the CNS inflammatory status in GT-treated mice, further suggesting an improvement in the neurological phenotype.^46^

Pre-clinical studies have shown that neonatal interventions with donor cells are highly effective in correcting the bone phenotype in MPS-I, with treatment during the first months of life being associated with the prevention of skeletal disease progression compared to later intervention.^6^^,^^50^^,^^51^ First, we observed that GT-treated mice displayed a reduced HS signal in both the bone and the growth plate. Although this result is not quantitative, it provides supporting evidence for the efficacy of neonatal treatment on the skeletal tissue, given the HS relevance in skeletal disorders associated with MPS-I. Furthermore, micro-CT analysis of femurs demonstrated improvement in bone architecture both at cortical and trabecular bone after neonatal GT, even though reduced femur length was retained. Although we did not focus on growth plates in this study, we cannot exclude that the GT approach does not affect, either completely or partially, the compromised chondrocyte phenotypic plasticity and the endochondral ossification process, that are known to be affected in MPS-IH.^52^ However, in spite of the overall skeletal improvement, we did not observe any correlation between PB IDUA levels and skeletal correction, differently from the data reported by Visigalli et al.^13^ Surprisingly, histomorphometric analysis showed that osteoclast parameters (N.Oc/BPm and Oc.S/BS) remained increased compared to WT also after GT. Further studies are needed to explain this result. It is known that GAGs, including DS and HS, inhibit the collagenolytic activity of cathepsin K,^53^^,^^54^ and that impaired osteoclast function is very important in the pathogenesis of the MPS-IH skeletal disease.^54^ Thus, it is possible that neonatal GT induced some functional recovery in the resorption activity of individual osteoclasts that was sufficient to ensure adequate bone remodeling.

This work contributes significantly to the field of MPSs treatment by evaluating the efficacy of *ex vivo* GT performed in newborns using a representative pre-clinical model of MPS-I. In particular, the study investigated the ability of neonatal GT to provide multisystemic correction, focusing on tissues that are not adequately corrected by currently available treatments.^55^^,^^56^ Treated mice showed a significant reduction in GAG accumulation, with no evidence cytoplasmic vacuolization in the analyzed visceral organs. Similarly, the altered skeletal phenotype showed improvements at radiographic and histological levels post-treatment. Considering the CNS impairment, an increase in IDUA activity accompanied by a significant reduction of vacuolization in the cortex of GT-treated mice was observed. However, no supraphysiological enzyme levels were detected, and complete GAG clearance was not achieved. The therapeutic results achieved with the neonatal *ex vivo* GT were compared to untreated WT controls in terms of metabolic and functional parameters, posing the base for the future preclinical proof of concept study and clinical translation of our neonatal therapeutic strategy. Including a control group of MPS-I mice GT treated as adults and a control group to monitor cell engraftment would strengthen future studies and deepen our understanding of the importance of treatment timing in MPS-I therapy. The wider implementation of newborn screening programs holds promise for translating the approach of neonatal transplantation of autologous GT-modified progenitor cells into experimental clinical trials.^51^^,^^57^^,^^58^ This approach, combined with early genome sequencing in newborns to detect disease-causing mutations, can potentially prevent disease progression. Additionally, performing GT in the neonatal period may reduce the risk of developing an immune response against the transgenic protein. Selection of the best preparative conditioning regimen in this early phase of life will be critical to minimize long-term side effects. Using neonatal cells for *ex vivo* GT in a clinical setting still presents challenges, particularly with respect to obtaining sufficient stem cells for transplantation. However, specific characteristics of the developing fetal hematopoietic system may offer advantages, including increased proliferative capacity, expansion of the stem cell pool, and the relatively low number of gene-modified stem cells required to achieve the therapeutic effect.^59^ Specific protocols to employ cryopreserved umbilical cord blood hematopoietic stem cells and to genetically modify them need to be optimized and validated to ensure clinical feasibility. Additionally, refinements of all settings, from the collection and transduction of HSCs to quality controls, are needed through the whole process to enable rapid patient infusion. AAV GT represents an alternative treatment option for neonatal MPS-I patients that could be applied fast after diagnosis. Further investigation is critical to counteract the immunological response and the risk of insertional mutagenesis, and to improve the transient efficacy and targeting of affected tissues.^60^

Finally, the positive results obtained in MPS-I represent a proof of principle to translate similar neonatal GT approaches in the context of other lysosomal storage diseases, providing confirmation of the effectiveness of an extremely early intervention for these disorders and offering the perspective of a potential therapeutic opportunity.

## Materials and methods

### Mice

*Idua*^−/−^ mice were acquired by the Fondazione San Raffaele del Monte Tabor animal facility as a kind gift from Prof J. M. Heard and inter-crossed to obtain an inbred strain.^13^^,^^20^ This MPS-I animal model is based on an immunocompetent C57BL/6 background. The colony was maintained through on-site matings between heterozygous mice, with subsequent selection of homozygotes by genotyping of tail or ear samples. All procedures involving animal handling and care were conducted in accordance with institutional guidelines and approved by the Organismo Preposto al Benessere Animale (OPBA) of the Fondazione San Raffaele del Monte Tabor (IACUC 759/1265), in compliance with national laws and policies (permit number 19/2017-PR from the Italian Ministry of Health).

### *In vitro* transduction procedure

BM cells were collected from the long bones of 8–10 weeks old *Idua*^−/−^ C57BL/6 mice by flushing. After lysis with ammonium chloride (ACK) lysing buffer (STEMCELL Technologies, Vancouver, British Columbia, Canada), BM cell populations were enriched in progenitor cells using the anti-Sca-1 MicroBead Kit (Miltenyi Biotech, Bergisch Gladbach, Germany), according to the manufacturer’s instructions. The enriched cells were cultured at the concentration of 1 × 10^6^ cells/mL in StemSpan SFEM (STEMCELL Technologies) supplemented with 1% glutamine, 1% Pen/Strep, 100 ng/μL human Fms-related tyrosine kinase 3 ligand (hFlt3-L), 100 ng/μL murine stem cell factor, 50 ng/μL murine thromobopoietin (TPO), and 20 ng/μL human interleukin 3 (Peprotech, Cranbury, NJ, USA). For LV transduction, hematopoietic progenitor cells were transduced overnight with an IDUA-LV vector (Figure S2A, vector titer: 2.4 × 10^9^ transducing units per mL) at different MOIs (0, 10, 20, 40, 60, and 80) added in the culture medium. For *in vivo* experiments, cells were transduced at an MOI of 40. The LV vector contain the human phosphoglycerate kinase promoter to drive the expression of the *IDUA* gene, as previously described.^13^ Cells were suspended in PBS (Corning, Corning, NY, USA) 12–16 h after infection for transplantation. A portion of the cells was maintained in LC for 14 days for VCN assay, evaluation of IDUA expression and for CFUs analysis. At days 0 and 14, cells were partially stained with anti-mouse Ly-6A/E (Sca-1) APC (D7, Invitrogen, Waltham, MA, USA) for monitoring the percentage of progenitor cells over time; samples were analyzed using a FACSCanto II flow cytometer with FACS Diva software (BD Biosciences, Franklin Lakes, NJ, USA) and FlowJo (Ashlans, OR, USA).

### Mice transplantation and experimental procedures

As outlined in the experimental procedure detailed in Figure 2D, C57BL/6-MPS-I Ly5.2 pups (1–2 days old) received busulfan (20 mg/kg, intraperitoneal injection) for preparatory conditioning regimen, as already described by our group.^5^^,^^6^^,^^11^ MPS-I Ly5.2 BM-derived hematopoietic progenitor cells were cultured overnight in the presence of the vector, and subsequently transplanted via a single injection into the temporal vein, within 24 h post-conditioning. The cell dose administered was 1 × 10^5^ cells/50 μL/pup. Untreated MPS-I and WT mice were included as control groups for comparison and mice of both sexes were equally represented. PB samples were collected monthly via tail vein bleeding and blood samples were then lysed with ACK buffer. Following storage, sample pellets were analyzed for IDUA activity and VCN evaluations until the time of sacrifice at 32 weeks, as further described in the following text.

Concerning the metabolic characterization, untreated young MPS-I mice were sacrificed, and the liver, spleen, lungs, kidneys, and heart were harvested without prior tissue perfusion. The adult MPS-I and WT mice (8–10 weeks of age) were compared in terms of IDUA activity and GAG storage. Assays were performed in visceral organs as further described in the following text.

### CFU assay

To evaluate the functionality of progenitor cells, 2.5 × 10^3^ *Idua*^−/−^ BM-derived progenitor cells or 6 × 10^4^ total BM cells isolated from mice at sacrifice were resuspended respectively in 2.5 or 3 mL MethoCult GF M3434 methylcellulose-based medium (STEMCELL Technologies) and cultured for 14 days at 37°C and 5% CO_2_. Hematopoietic colonies were counted to evaluate the proliferating ability of progenitor cells under *in vitro* and LC conditions.

### Vector copy number assay

Genomic DNA was extracted from cellular pellets collected after 14 days of culture and from visceral organs obtained at sacrifice from treated mice using the QIAamp DNA Mini Kit (QIAGEN, Hilden, Germany), according to the manufacturer’s instructions. To determine the number of vector copies per diploid genome of gene-corrected cells, droplet digital PCR technology was employed, detecting the DNA sequence on the LV vectors’ common packaging signal region (human immunodeficiency virus system, HIV system) and utilizing the DNA sequence of mouse semaphorin as an endogenous control, as previously described.^31^ DNA extracted from UT cells or from WT animals were also evaluated as controls.

### Anti-IDUA IgG antibodies concentration in sera

Serum was obtained from PB of both untreated and treated mice after coagulation at room temperature (RT) and centrifugation at 3,000 rpm for ten minutes. Serum samples were then stored at −20°C until evaluation of anti-IDUA antibody formation. As described by Squeri et al.,^61^ an ELISA assay was performed on diluted serum samples (1:100). The experimental procedure includes overnight coating of the plate with recombinant human IDUA (2 μg/mL), blocking with PBS-1% BSA (Roche, Basel Switzerland), incubation of duplicate serum samples, followed by secondary antibody addition for 2 h and rapid reaction with ophenylenediamine dihydrochloride and H_2_O_2_. The anti-IDUA IgG concentration (μg/mL) was determined by measuring absorbance at 492 nm with SkaltRE Multiskan go spectrophotometer (v.3.2, Thermo Fisher Scientific, Waltham, MA, USA) and quantified by comparison with murine IgGs at known concentrations.

### IDUA activity assay

IDUA activity was evaluated in LV-transduced cells after 14 days of LC, in PB mononuclear cells obtained by lysing PB samples with ACK buffer, and in BM cells obtained after tibia flushing at sacrifice. Samples were stored at −20°C until analyses were performed. Cellular pellets were resuspended in saline solution and lysed using freezing and thawing cycles in addition to sonication (UTR200, Hielscher Ultrasonics, Teltow, Germany). The liver, spleen, kidneys, lungs, heart, and brain samples obtained at the endpoint were stored at −80°C after freezing with dry ice, as previously described.^6^^,^^11^ Visceral organs were lysed for one hour at 4°C in NaCl 0.9% solution containing Triton X-100 (0.2%, Sigma-Aldrich) and protease inhibitor cocktail (PIC, 100×, Thermo Fisher Scientific). After pelleting the homogenates of organs, the Pierce BCA assay (Thermo Fisher Scientific) was performed to quantify proteins in the supernatants. For the IDUA assay, 5 μg of protein samples were incubated with the fluorogenic substrate 4-methylumbelliferyl-alpha-L-iduronide (4 MU-I, Glycosynth, Warrington, England) in a sodium formate solution (0.1 M [pH 3.2]), with D-saccharic acid 1,4-lactone (0.1 mM). After incubation for 1 h at 37°C, carbonate buffer (0.5 M [pH 10.7]) was added to stop the reaction, and IDUA activity was evaluated by measuring fluorescence using the FLUOstar omega plate reader (BMG LABTECH, Ortenberg, Germany, 365 nm excitation/488 nm emission). IDUA activity in each sample was calculated using a calibration curve of 4-methylumbelliferone (4-MU, Sigma-Aldrich).

### Quantification of GAG accumulation in visceral organs and plasma

After dry ice freezing and storage at −80°C, the liver, spleen, heart, lungs, and kidneys of 32-week-old mice were digested overnight in the presence of papain (Sigma Aldrich) at 65°C. Following the digestion process, homogenates of the organs were pelleted, and the Pierce bicinchoninic acid (BCA) protein assay kit (Thermo Fisher Scientific) was performed to quantify proteins in the supernatants. A quantitative dye-binding assay (Blyscan Glycosaminoglycan Assay; Biocolor Ltd., Carrickfergus, United Kingdom) was employed for quantifying GAG storage in visceral organs, according to the manufacturer’s instructions. Absorbance was measured at 620 nm using aSkaltRE Multiskan go spectrophotometer (v.3.2, Thermo Fisher Scientific), and the output was indicated as μg GAGs/mg protein.

For GAG quantification in plasma, PB harvested prior to sacrifice was collected in ethylenediaminetetraacetic acid (EDTA) and stored at −80°C after centrifugation (587 g × 10 min). The experimental procedure was carried out as previously described, utilizing liquid chromatography triple quad mass spectrometry (LC-MS/MS) to calculate disaccharides concentrations after polysaccharides digestion.^11^

### Histopathology, radiographs, and bone morphometry quantification

At the experimental endpoint, the spleen, liver, kidneys, lungs, heart, and cerebral cortex were collected, without prior tissue perfusion, and ¼ of each organ was fixed in a phosphate buffer (0.12 M) with 4% paraformaldehyde and 2% glutaraldehyde, as previously described.^11^ Osmium tetroxide (1%) was then added for additional fixation, and the samples were left overnight at 60°C after embedding in Epon (Sigma-Aldrich) for transverse section cutting (1 micron). For the analysis of the Purkinje cell layer, cerebellum sections at sacrifice were fixed in a phosphate buffer (0.12 M) with 4% paraformaldehyde, followed by the addition of 30% sucrose for storage at 4°C. Toluidine blue staining was performed to analyze morphological differences between the experimental groups. Images were taken at 20× magnification (for the kidney, heart, spleen, cerebellum, and cortex) and 40× magnification (for the lungs, and renal glomeruli). Ten random images from 3 to 4 different mice per group were scanned to quantify cytoplasmic vacuolization across different compartments. GAG storage was expressed as the percentage of vacuolation over the total section area considered or the total section for renal glomeruli. Cryosections of cerebellum samples (thickness = 10 μm) were obtained after embedding in optimal cutting temperature compound and liquid nitrogen freezing. Staining procedure was performed as previously described,^11^ using rabbit anti-Iba-1 antibody (FUJIFILM Wako Pure Chemical Corporation, Chuo-ku, Osaka, Japan; 1:200; 1 h at RT) and FITC-conjugated secondary antibody (Southern Biotechnology Associates Inc., Birmingham, AL, USA, 1:100). Ten random images from 3 to 4 different mice per group were quantified.

Skeletal histological analyses were performed on one femur per mouse at the endpoint. The femurs were decalcified in 10% EDTA (Sigma-Aldrich) and embedded in paraffin. Paraffin sections (4 μm thick) were rehydrated and used for standard histology with hematoxylin and eosin (H&E) stain and for tartrate-resistant-acid-phosphatase (TRAP) histochemistry to highlight mono- and multinucleated osteoclasts. TRAP staining was performed dissolving 50 mg of naphthol AS-BI phosphate (disodium 6-bromo-3-[(2-methoxyphenyl)carbamoyl]naphthalen-2-yl phosphate) in 4 mL of N,N-dimethylformamide, adding 4 mL of acetate buffer and 92 mL of distilled water and 150 mg of tartaric acid and 30 mg of Fast Garnet. Slides were incubated in this final working solution for 30 min at 37°C. All reagents were purchased from Sigma-Aldrich. Images were obtained with the Zeiss Axiophot microscope.

Histomorphometry analysis was performed on 4–10 20× fields of the metaphyseal area of mice femurs. Measurements of osteoblast number per bone perimeter (N.Ob/B.Pm; N/mm), osteoblast surface per bone surface (Ob.S/BS; %), osteoclast number per bone surface (N.Oc/B.Pm; N/mm) and osteoclast surface per bone surface (Oc.S/BS; %), were all performed with ImageJ software (Rasband, W.S., ImageJ, NIH, Bethesda, MD, USA).

For immunolocalization of HS in bones, 3-μm thick paraffin-embedded sections were immunostained with the mouse monoclonal antibody anti-HS (370255, AmsBio, Oxfordshire, UK; 1:100; 2 h at RT). To prevent nonspecific antibody binding, blocking with 10% bovine serum albumin in PBS was performed. Following primary antibody incubation, slides were repeatedly washed in PBS and incubated with biotin-conjugated rabbit anti-mouse IgG (P0260, Agilent) for 30 min at RT in a humidified chamber. After additional PBS washes, slides were incubated with streptavidin-conjugated horseradish peroxidase (P0397, Agilent) for 30 min at RT. The peroxidase reaction was developed using a 3,3′-diaminobenzidine (DAB) substrate kit (SK-4105, Vector Laboratories, Newark, CA, USA).

Radiographic analyses were performed on femurs of mice at sacrifice, which were previously fixed in 4% formaldehyde. The Faxitron System (MX-20, Hologic, Marlborough, MA, USA) was used for radiographs with 30 kV energy, and X-ray film was developed using the Model M35A developer (Eastman Kodak Corporate, Rochester, NY, USA). Measurements of bone width and length were conducted using ImageJ software (Rasband, W.S., ImageJ, NIH, Bethesda, MD, USA).

Micro-CT analyses were performed on the right femurs of WT, MPS-I untreated and treated mice. The femurs were wrapped in gauze soaked with 0.9% saline and placed in vials containing saline solution. Samples were analyzed using the SkyScan 1175 system (Bruker, Billerica, MA, USA), with scan settings of 80 kV voltage and 50 μA current. Morphometric evaluations were performed with Bruker CT-Analyzer (CTAn) software after reconstruction of cortical and trabecular bone regions. Parameters such as bone volume over total volume (BV/TV; %), average trabecular thickness (AVG Tb Th; mm), bone mineral density (BMD; gHA/ccm), total area (mm^2^), bone area (mm^2^), the bone area over the total area (BA/TA; %), and average cortical thickness (AVG Cort Thick; mm) were analyzed.

### Statistical analyses

The analyses were performed using GraphPad Prism v.10 software (GraphPad Prism, San Diego, CA, USA). For both *in vitro* and *in vivo* experiments, data have been analyzed using non-parametric analyses, considering the small sample size (*n* < 10). Non-parametric one-way ANOVA test with Kruskal-Wallis test was performed for multiple comparisons to a control group, as indicated in the figure legends. The non-parametric Mann-Whitney test has been employed for comparison between two groups. *p* < 0.05 has been considered statistically significant. The Pearson test was used to assess linear correlation. Statistically significant differences were considered as follows: ∗*p* < 0.05, ∗∗*p* < 0.01, ∗∗∗*p* < 0.001, and ∗∗∗∗*p* < 0.0001. When not specified, comparisons between groups were not significant.

## Data availability

For original data, please contact marta.serafini@unimib.it.

## Acknowledgments

We thank Isabella Azario for technical support and Alessandro Nonis from the Centro Universitario di Statistica per le Scienze Biomediche (CUSSB, San Raffaele Hospital) as a consultant for the statistical analyses. This work was supported by 10.13039/501100003042Else Kröner-Fresenius-Stiftung, Germany EKF prize for Medical Research 2020 to A. Aiuti.

## Author contributions

G.D.P., L.S., and A.P. performed research, analyzed data, and wrote the paper, G.D., S.D., S.K., L.P., and A. Annoni performed research and analyzed data; A.C., M.R., S.G., and S.C. analyzed data, interpreted data, and edited the manuscript; A.N., A.B., A.Q., S.T., and A. Aiuti edited the manuscript; M.E.B. and M.S. designed research, interpreted data, and wrote the paper.

## Declaration of interests

The authors declare no competing interests.

## Supplemental information

Document S1. Figures S1–S3

Document S2. Article plus supplemental information
