# Supplementary material for: Neonatal gene therapy effectively prevents disease manifestations in a murine model of Mucopolysaccharidosis type I
Source: Mol Ther Methods Clin Dev. 2025 Jul 30;33(3):101544. doi: 10.1016/j.omtm.2025.101544 (PMC12357110; doi:10.1016/j.omtm.2025.101544)
Supplement: Document S1. Figures S1–S3 [file mmc1.pdf]

OMTM, Volume *33*

Supplemental information

Neonatal gene therapy effectively prevents disease manifestations in a murine model of Mucopolysaccharidosis type I

Giada De Ponti, Ludovica Santi, Giorgia Dina, Alice Pievani, Samantha Donsante, Mara Riminucci, Alessandro Corsi, Shaukat Khan, Laura Passerini, Andrea Annoni, Silvia Gregori, Stefania Crippa, Andrea Biondi, Angelo Quattrini, Shunji Tomatsu, Alessandro Aiuti, Maria Ester Bernardo, and Marta Serafini

SUPPLEMENTAL INFORMATION

Figure S1

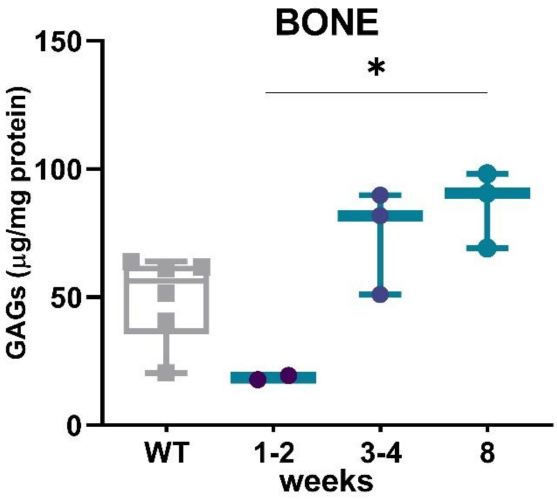

Figure S1. Quantification of GAG accumulation in bones of untreated ageing MPS-I mice. GAG storage was evaluated in the bones of WT mice of different ages (n=6, represented as gray plots in the graph) and MPS-I mice at 1-2, 3-4, and 8 weeks of age (n=2-3, for each age). Each data point represents an individual mouse, while bars indicate the median value. *P≤0.05 by non-parametric one-way ANOVA with Kruskal-Wallis test.

Figure S2

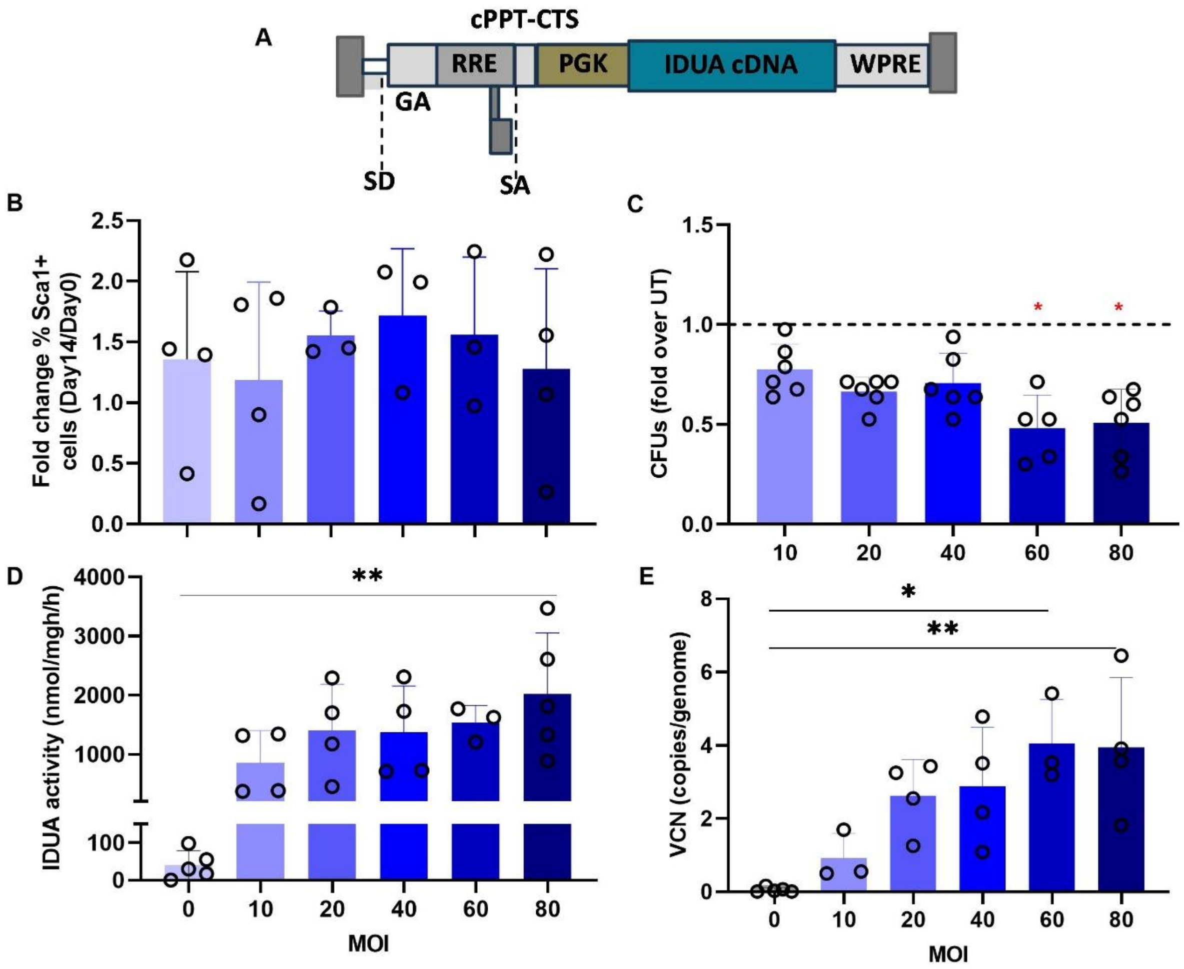

Figure S2. ***In vitro* setting.** (**A**) Schematic representation of the lentiviral transfer vector encoding the IDUA cDNA. (**B**) Sca-1 positivity over time was assessed by measuring expression levels on day 0 and 14 post-infection at varying MOI ranging from 0 to 80 (n≥3, for each MOI), and expressing the results as fold-change relative to plating day values. (**C, D, E**) Analysis of clonogenic capacity, IDUA activity and vector integration (VCN) of the same cells (n≥3, for each MOI). Each bar represents the mean value (SD). *p≤0.05; **p≤0.01; ***p≤0.001by non-parametric one-way ANOVA with Kruskal-Wallis test. Red asterisks in panel C indicate significance compared to MOI 0 condition (represented by the dotted line).

Figure S3

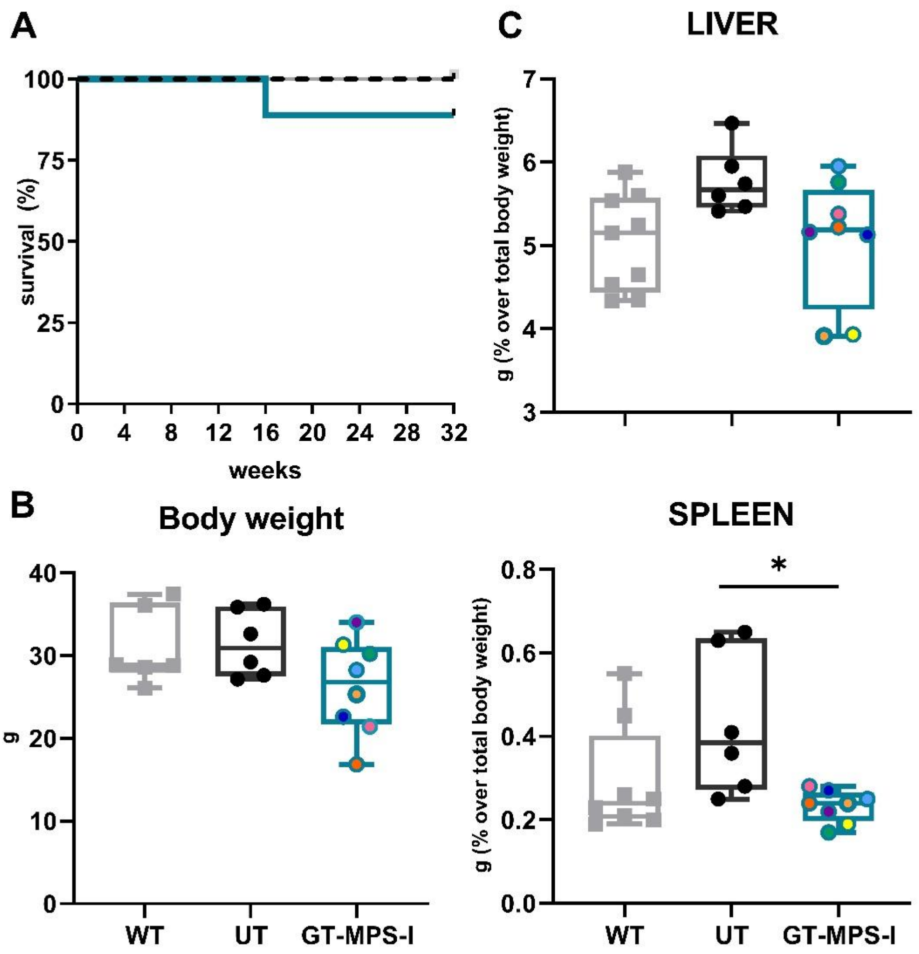

Figure S3. Treatment tolerability assessment. (**A**) Survival curve of wild type (grey plots), untreated (black plots) and treated MPS-I (blue plots) over time until endpoint. (**B**) Total body weight comparison between wild type (grey plots), untreated (black plots) and treated MPS-I mice (blue plots) at the study endpoint of 32 weeks. (**C**) Organ-to-body weight ratios for liver and spleen in the same animal groups. Each mouse is represented with a data point, while the bar indicates the median value. *$p \leq 0.05$ by non-parametric one-way ANOVA with Kruskal-Wallis test.
